# 7-(4-Chloro­phen­yl)-9-phenyl-7*H*-pyrrolo[3,2-*e*]tetra­zolo[1,5-*c*]pyrimidine

**DOI:** 10.1107/S160053681000485X

**Published:** 2010-02-13

**Authors:** Rina D. Shah, Mukesh M. Jotani, Jerry P. Jasinski

**Affiliations:** a Department of Chemistry, M.G. Science Institute, Navrangpura, Navrangpura, Ahmedabad, Gujarat, 380 009, India; b Department of Physics, Bhavan’s Sheth R.A. College of Science, Ahmedabad, Gujarat, 380 001, India; c Department of Chemistry, Keene State College, 229 Main Street, Keene, NH 03435-2001, USA

## Abstract

In the title compound, C_18_H_11_ClN_6_, the pyrrole, pyrimidine and tetra­zole rings form a nearly planar fused trihetrocyclic system with an r.m.s. deviation of 0.0387 (13) Å, to which the 4-chloro­phenyl group and the phenyl group are substituted at the 7 and 9 positions, respectively. The dihedral angles between the pyrrole ring and the 4-chloro­phenyl and phenyl rings are 32.1 (4) and 7.87 (7)°, respectively. In the crystal, weak inter­molecular C—H⋯N and C—H⋯Cl hydrogen bonds link the mol­ecules into a layer parallel to the (001) plane. The layers are further connected by π–π stacking inter­actions [centroid–centroid distances: 3.8413 (8) and 3.5352 (8) Å]. Intra­molecular C—H⋯N hydrogen bonds are also present.

## Related literature

For nucleophilic substitution reactions, see: Augustine & Agrawal (2005 ▶); Dave & Shah (1998 ▶); Desai & Shah (2006 ▶). For phase-transfer catalysis techniques, see: Hartwig (1997 ▶, 1998 ▶); Frost & Mendoncua (1998 ▶). For eductive ring cleavage reactions, see: Martarello (2001 ▶); Gangjeea *et al.* (2005 ▶). For the biological activity of fused tetra­zolopyrimidines, see: Shishoo & Jain (1992 ▶); Desai & Shah (2006 ▶). For a related structure, see: Jotani *et al.* (2010 ▶). For graph-set motifs, see: Bernstein *et al.* (1995 ▶). For MOPAC PM3 calculations, see: Schmidt & Polik (2007 ▶).
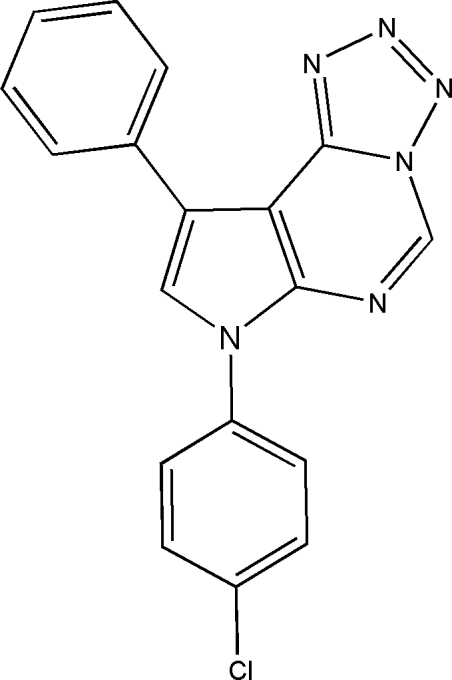


## Experimental

### 

#### Crystal data

C_18_H_11_ClN_6_
*M* *_r_* = 346.78
Monoclinic, 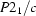

*a* = 11.8335 (3) Å
*b* = 17.4200 (5) Å
*c* = 7.4094 (2) Å
β = 91.129 (1)°
*V* = 1527.07 (7) Å^3^
*Z* = 4
Mo *K*α radiation
μ = 0.26 mm^−1^
*T* = 293 K
0.40 × 0.20 × 0.15 mm

#### Data collection

Bruker APEXII CCD diffractometer
Absorption correction: multi-scan (*SADABS*; Sheldrick, 1999 ▶) *T*_min_ = 0.941, *T*_max_ = 0.961
22397 measured reflections
5544 independent reflections
3946 reflections with *I* > 2σ(*I*)
*R*_int_ = 0.024

#### Refinement

*R*[*F* ^2^ > 2σ(*F* ^2^)] = 0.046
*wR*(*F* ^2^) = 0.138
*S* = 1.00
5544 reflections
226 parameters
H-atom parameters constrained
Δρ_max_ = 0.36 e Å^−3^
Δρ_min_ = −0.24 e Å^−3^

### 

Data collection: *APEX2* (Bruker, 2004 ▶); cell refinement: *APEX2* and *SAINT* (Bruker, 2004 ▶); data reduction: *SAINT* and *XPREP* (Bruker, 2004 ▶); program(s) used to solve structure: *SIR97* (Altomare *et al.*, 1999 ▶); program(s) used to refine structure: *SHELXL97* (Sheldrick, 2008 ▶); molecular graphics: *PLATON* (Spek, 2009 ▶); software used to prepare material for publication: *PLATON*.

## Supplementary Material

Crystal structure: contains datablocks I. DOI: 10.1107/S160053681000485X/is2522sup1.cif

Structure factors: contains datablocks I. DOI: 10.1107/S160053681000485X/is2522Isup2.hkl

Additional supplementary materials:  crystallographic information; 3D view; checkCIF report

## Figures and Tables

**Table 1:** Hydrogen-bond geometry (Å, °)

*D*—H⋯*A*	*D*—H	H⋯*A*	*D*⋯*A*	*D*—H⋯*A*
C8—H8⋯N4^i^	0.93	2.54	3.393 (2)	153
C5—H5⋯Cl1^ii^	0.93	2.82	3.6045 (15)	143
C12—H12⋯N6	0.93	2.32	3.191 (2)	155
C18—H18⋯N2	0.93	2.48	2.979 (2)	114

Symmetry codes: (i) 
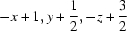
; (ii) 
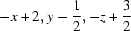
.

**Fig. 1.**
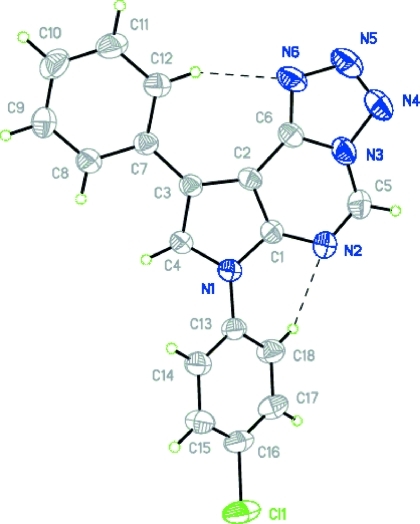
The molecular structure of (I), showing the atom labelling scheme and 50% probability displacement ellipsoids. The dashed line represents a weak intramolecular C—H···N hydrogen bond.

**Fig. 2.**
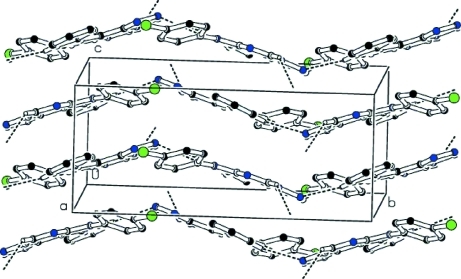
Crystal packing of (I), viewed down the a axis showing weak intramolecular C—H···N hydrogen bond interactions.

**Fig. 3.**
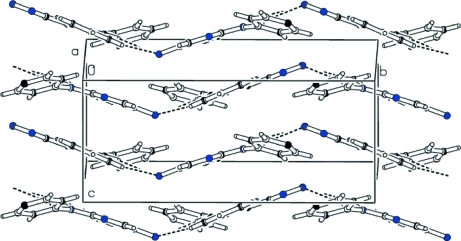
Crystal packing of (I), showing weak C—H···N and C—H···Cl intermolecular interactions. H atoms not involved in hydrogen bonding have been omitted.

**Fig. 4.**
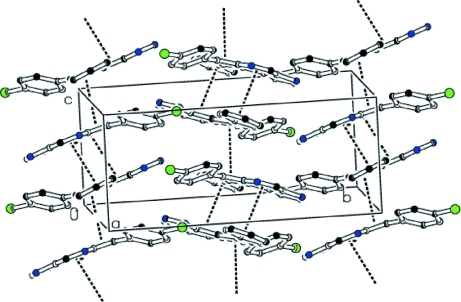
The molecular packing of (I), showing π—π stacking interactions along the c axis and forming a chain of molecules along [0 0 1].

## supplementary crystallographic information

### Comment

Nucleophilic substitutions are key reactions having enormous applications in the field of organic synthetic chemistry (Augustine & Agrawal, 2005; Desai & Shah, 2006). Synthesis of tetrazolopyrrolopyrimidines involves studies of various nucleophilic displacements (Dave & Shah, 1998; Desai & Shah, 2006) such as chlorination, azidolysis and amination in fused pyrimidines. Phase Transfer Catalysis (PTC), an environmental benign technique (Hartwig, 1997, 1998), offers many advantages over conventional methodologies *viz* use of non-polar solvents, reduced reaction temperature and reaction time, suppression of side products, high yield, replacement of hard bases and easy work up. Strategies involving eco friendly phase transfer catalysis with nucleophilic displacements have always been of great interest. Generally, amination of 4-chloroazines uses harsh reaction conditions (Hartwig, 1998; Frost & Mendoncua, 1998) while fused tetrazole are found to posess latent amino functionality giving facile amination (Shishoo & Jain, 1992; Desai & Shah, 2006). Reductive ring cleavage of tetrazolo[1,5-*c*]pyrrolo[3,2-*e*]pyramidines results in the formation of 4-aminopyrrolo[2,3-*d*]pyrimidines, the landmarks of pharmaceutics (Desai & Shah, 2006; Martarello, 2001; Gangjeea *et al.*, 2005). In addition a variety of biological activities have been attributed to fused tetrazolopyrimidines (Shishoo & Jain, 1992; Desai & Shah, 2006). In view of the importance of these compounds, we report the crystal structure of title compound (I).

The title compound, C_18_H_11_ClN_6_, (I), is composed of a triheterocycle ring system resulting from the fusion of a benzene and 4-chlorobenzene substituted pyrrole and a tetrazole ring to a pyrimidine ring in a nearly planar fashion (Fig. 1). The r. m. s. deviation of atoms of the fused triheterocycle ring system from the mean plane through it is 0.0387 Å. The bond lengths and angles of fused tetrazole and pyrrole ring in (I) are normal and similar to those observed in a similar structure (Jotani *et al.*, 2010). The planarity of each of the five rings (tetrazole, pyrrole, pyrimidine, 4-chlorophenyl and benzene) is confirmed by the r.m.s. deviation values (0.0017, 0.0043, 0.0107,0.0059 and 0.0017 Å), respectively. The dihedral angle between the least squares planes of the tetrazole and pyrimidine rings fused with the pyrrole ring are 2.61 (7) and 5.42 (8)°. The dihedral angles between the mean planes of the ortho-substituted 4-chlorophenyl and benzene rings with the pyrrole ring are 32.1 (4) and 7.87 (7)°, respectively.

The crystal structure is supported by weak C—H···N intramolecular (Fig. 2 & 3) and weak C—H···Cl and C—H···N intermolecular (Fig. 3) interactions which link the molecules into a layer parallel to the (001) plane (Table 1). The weak C—H···N intramolecular interactions forms pseudo R2^2^(8)S(6) and R2^2^(8)S(7) graph-set motifs (Fig. 1, Table 1) (Bernstein *et al.*, 1995) between the benzene and triheterocycle rings and between the 4-chlorobenze and triheterocycle rings, respectively. In addition, crystal packing is also supported by two weak π–π stacking interactions (Fig. 4). One is between the centroids of two pyrrole rings [*Cg*1—*Cg*1(1-*x*, -*y*, 2-*z*) 3.841 (2) Å; slippage 1.420 Å; *Cg*1 = N1/C1–C4]. The second is between the centroids of a pyrimidine (*Cg*3) and phenyl (*Cg*4) ring [*Cg*3—*Cg*4(1-*x*, -*y*, 1-*z*) 3.535 (2) Å; *Cg*3 = N2/C1/C2/C6/N3/C5; *Cg*4 = C7–C12].

After a geometry optimized MOPAC PM3 computational calculation (Schmidt & Polik, 2007) on (I), in vacuo, the angle between the mean planes of the pyrimidine and tetrazole groups become completely planar with the pyrrole ring in the local minimized structure. The dihedral angle between the mean planes of the ortho-substituted 4-chlorophenyl and benzene rings and the now completely planar triheterocycle group becomes 42.17 and 41.70°, respectively. The separation of the H4···H12 (2.305 Å) and H4···H18 (2.103 Å) atoms between the pyrrole ring and the 4-chlorophenyl and benzene rings before the calculations changed to 2.492 and 2.588 Å, respectively, after the calculation showing how the crystal packing effects significantly determine the twist of both the 4-chlorophenyl and benzene rings. In addition, the C3–C7 and N1–C13 bond lengths changed from 1.4696 (16) and 1.4221 (15) Å to 1.455 and 1.442 Å, respectively. It is clear that the collective action of these intramolecular, intermolecular and π-π stacking interactions interactions significantly influence the twist angles for the molecule in this crystal.

### Experimental

The title compound is synthesized by three different routes and Phase Transfer Catalysis is novel among them. To a well stirred mixture of 5-phenyl-7-(4-chlorophenyl)-4-chloro-7*H*-pyrrolo[2,3-*d*]pyrimidine (5 mmol) and Aliquat 336 (0.5 mmol) in toluene (25 ml) was added sodium azide (6 mmol) in water (5 ml). The reaction mixture was stirred under reflux condition for 1–1.5 h. Thereafter, the two phases were separated. The aqueous phase was extracted with toluene and combined organic layers were washed with water. The excess of solvent was distilled under reduced pressure. The obtained solid was filtered, dried, and crystallized from 1,4-dioxane.

### Refinement

H atoms were placed in idealized positions (C—H = 0.93—0.98 Å) and constrained to ride on their parent atoms with *U*_iso_(H) = 1.2*U*_eq_(C).

### Crystal data

**Table:**

C_18_H_11_ClN_6_	*F*(000) = 712
*M**_r_* = 346.78	*D*_x_ = 1.508 Mg m^−^^3^
Monoclinic, *P*2_1_/*c*	Mo *K*α radiation, λ = 0.71073 Å
Hall symbol: -P 2ybc	Cell parameters from 500 reflections
*a* = 11.8335 (3) Å	θ = 1.8–30.0°
*b* = 17.4200 (5) Å	µ = 0.26 mm^−^^1^
*c* = 7.4094 (2) Å	*T* = 293 K
β = 91.129 (1)°	Plate, colorless
*V* = 1527.07 (7) Å^3^	0.40 × 0.20 × 0.15 mm
*Z* = 4	

### Data collection

**Table:**

Bruker APEXII CCD diffractometer	5544 independent reflections
Radiation source: fine-focus sealed tube	3946 reflections with *I* > 2σ(*I*)
graphite	*R*_int_ = 0.024
φ and ω scans	θ_max_ = 32.6°, θ_min_ = 2.1°
Absorption correction: multi-scan (*SADABS*; Sheldrick, 1999)	*h* = −17→17
*T*_min_ = 0.941, *T*_max_ = 0.961	*k* = −26→26
22397 measured reflections	*l* = −10→11

### Refinement

**Table:**

Refinement on *F*^2^	Primary atom site location: structure-invariant direct methods
Least-squares matrix: full	Secondary atom site location: difference Fourier map
*R*[*F*^2^ > 2σ(*F*^2^)] = 0.046	Hydrogen site location: inferred from neighbouring sites
*wR*(*F*^2^) = 0.138	H-atom parameters constrained
*S* = 1.00	*w* = 1/[σ^2^(*F*_o_^2^) + (0.066*P*)^2^ + 0.3891*P*] where *P* = (*F*_o_^2^ + 2*F*_c_^2^)/3
5544 reflections	(Δ/σ)_max_ = 0.001
226 parameters	Δρ_max_ = 0.36 e Å^−^^3^
0 restraints	Δρ_min_ = −0.24 e Å^−^^3^

### Special details

**Table:**

Experimental. Additionoal synthetic routes: A) A mixture of sodium azide (0.011 mole), ammonium chloride (0.011 mole) and 5-phenyl-7-(4-chlorophenyl)-4-chloro-7*H*-pyrrolo[2,3-*d*] pyrimidine (0.01 mole) in DMSO (20 ml) was stirred for for 2 h at 363 K to give the title compound which was crystallized from dioxane.(B) To a mixture of 5-phencyl-7-(4-chlorophenyl)-4-hydrazino-7*H*-pyrrolo[2,3-*d*]\ pyrimidine (0.01 mole) in acetic acid (40 ml) was added aqueous solution of sodium nitrite (20% *w*/*v*, 4.2 ml) in portions with stirring at 273–278 K and the reaction mixture was further stirred for 2 hr at the same temperature. Then it was diluted with cold water and the solid obtained was filtered, washed with water, sodium bicarbonate (20% *w*/*v*), followed by water, dried and crystallized from dioxane.
Geometry. All esds (except the esd in the dihedral angle between two l.s. planes) are estimated using the full covariance matrix. The cell esds are taken into account individually in the estimation of esds in distances, angles and torsion angles; correlations between esds in cell parameters are only used when they are defined by crystal symmetry. An approximate (isotropic) treatment of cell esds is used for estimating esds involving l.s. planes.
Refinement. Refinement of *F*^2^ against ALL reflections. The weighted *R*-factor *wR* and goodness of fit *S* are based on *F*^2^, conventional *R*-factors *R* are based on *F*, with *F* set to zero for negative *F*^2^. The threshold expression of *F*^2^ > σ(*F*^2^) is used only for calculating *R*-factors(gt) *etc*. and is not relevant to the choice of reflections for refinement. *R*-factors based on *F*^2^ are statistically about twice as large as those based on *F*, and *R*- factors based on ALL data will be even larger.

### Fractional atomic coordinates and isotropic or equivalent isotropic displacement parameters (Å^2^)

**Table:**

	*x*	*y*	*z*	*U*_iso_*/*U*_eq_	
Cl1	1.01669 (4)	0.26673 (3)	0.99565 (6)	0.06192 (15)	
N1	0.65304 (9)	0.04516 (6)	0.82381 (15)	0.0346 (2)	
N2	0.75894 (10)	−0.07074 (7)	0.78100 (17)	0.0426 (3)	
N3	0.65113 (11)	−0.17103 (7)	0.66593 (18)	0.0434 (3)	
N4	0.63580 (14)	−0.24253 (7)	0.5952 (2)	0.0590 (4)	
N5	0.52978 (14)	−0.24604 (8)	0.5529 (2)	0.0628 (4)	
N6	0.47357 (12)	−0.18044 (7)	0.5911 (2)	0.0504 (3)	
C1	0.66062 (10)	−0.03079 (7)	0.77751 (16)	0.0333 (2)	
C2	0.55269 (10)	−0.05656 (7)	0.72492 (16)	0.0318 (2)	
C3	0.47622 (10)	0.00643 (7)	0.74307 (16)	0.0312 (2)	
C4	0.54180 (10)	0.06642 (7)	0.80355 (17)	0.0345 (3)	
H4	0.5144	0.1153	0.8278	0.041*	
C5	0.75188 (13)	−0.14057 (9)	0.7264 (2)	0.0474 (3)	
H5	0.8164	−0.1711	0.7279	0.057*	
C6	0.55028 (11)	−0.13325 (7)	0.66188 (18)	0.0368 (3)	
C7	0.35371 (10)	0.01186 (7)	0.70905 (17)	0.0325 (2)	
C8	0.29678 (12)	0.07857 (8)	0.7572 (2)	0.0416 (3)	
H8	0.3368	0.1184	0.8122	0.050*	
C9	0.18247 (12)	0.08666 (9)	0.7248 (2)	0.0483 (3)	
H9	0.1463	0.1317	0.7580	0.058*	
C10	0.12136 (13)	0.02846 (10)	0.6436 (2)	0.0517 (4)	
H10	0.0440	0.0338	0.6224	0.062*	
C11	0.17620 (13)	−0.03772 (10)	0.5943 (2)	0.0502 (4)	
H11	0.1355	−0.0771	0.5385	0.060*	
C12	0.29125 (11)	−0.04658 (8)	0.6267 (2)	0.0411 (3)	
H12	0.3269	−0.0918	0.5931	0.049*	
C13	0.74236 (10)	0.09744 (7)	0.86362 (17)	0.0346 (3)	
C14	0.73102 (12)	0.17313 (8)	0.8092 (2)	0.0418 (3)	
H14	0.6669	0.1887	0.7445	0.050*	
C15	0.81516 (13)	0.22565 (9)	0.8513 (2)	0.0462 (3)	
H15	0.8073	0.2769	0.8178	0.055*	
C16	0.91070 (12)	0.20107 (9)	0.94355 (19)	0.0434 (3)	
C17	0.92339 (12)	0.12600 (10)	0.9961 (2)	0.0479 (3)	
H17	0.9887	0.1103	1.0575	0.057*	
C18	0.83840 (12)	0.07379 (9)	0.9570 (2)	0.0435 (3)	
H18	0.8458	0.0229	0.9935	0.052*	

### Atomic displacement parameters (Å^2^)

**Table:**

	*U*^11^	*U*^22^	*U*^33^	*U*^12^	*U*^13^	*U*^23^
Cl1	0.0518 (2)	0.0722 (3)	0.0619 (3)	−0.0327 (2)	0.00217 (17)	−0.0090 (2)
N1	0.0319 (5)	0.0306 (5)	0.0413 (5)	−0.0017 (4)	−0.0019 (4)	−0.0025 (4)
N2	0.0358 (5)	0.0407 (6)	0.0514 (7)	0.0053 (5)	0.0009 (5)	−0.0025 (5)
N3	0.0491 (6)	0.0278 (5)	0.0534 (7)	0.0045 (5)	0.0052 (5)	0.0004 (5)
N4	0.0687 (9)	0.0289 (6)	0.0794 (10)	0.0041 (6)	0.0055 (8)	−0.0073 (6)
N5	0.0726 (10)	0.0293 (6)	0.0864 (11)	−0.0019 (6)	−0.0023 (8)	−0.0084 (7)
N6	0.0558 (7)	0.0262 (5)	0.0689 (8)	−0.0050 (5)	−0.0055 (6)	−0.0034 (5)
C1	0.0339 (5)	0.0305 (6)	0.0355 (6)	0.0006 (4)	0.0010 (4)	0.0008 (5)
C2	0.0358 (6)	0.0258 (5)	0.0338 (6)	−0.0018 (4)	0.0004 (4)	0.0025 (4)
C3	0.0322 (5)	0.0266 (5)	0.0347 (6)	−0.0011 (4)	−0.0012 (4)	0.0024 (4)
C4	0.0332 (5)	0.0275 (6)	0.0426 (6)	0.0003 (4)	−0.0014 (5)	−0.0013 (5)
C5	0.0418 (7)	0.0414 (8)	0.0591 (9)	0.0095 (6)	0.0032 (6)	−0.0011 (6)
C6	0.0432 (6)	0.0273 (6)	0.0400 (6)	−0.0007 (5)	0.0010 (5)	0.0036 (5)
C7	0.0326 (5)	0.0297 (6)	0.0350 (6)	−0.0024 (4)	−0.0030 (4)	0.0040 (4)
C8	0.0384 (6)	0.0318 (6)	0.0543 (8)	−0.0001 (5)	−0.0047 (5)	0.0005 (6)
C9	0.0425 (7)	0.0422 (8)	0.0600 (9)	0.0098 (6)	−0.0062 (6)	0.0043 (7)
C10	0.0367 (7)	0.0613 (10)	0.0567 (9)	0.0046 (6)	−0.0117 (6)	0.0038 (7)
C11	0.0405 (7)	0.0531 (9)	0.0563 (9)	−0.0068 (6)	−0.0136 (6)	−0.0038 (7)
C12	0.0383 (6)	0.0375 (7)	0.0472 (7)	−0.0029 (5)	−0.0057 (5)	−0.0042 (6)
C13	0.0319 (5)	0.0348 (6)	0.0372 (6)	−0.0051 (5)	0.0002 (4)	−0.0026 (5)
C14	0.0372 (6)	0.0352 (7)	0.0527 (8)	−0.0035 (5)	−0.0047 (5)	−0.0006 (6)
C15	0.0467 (7)	0.0375 (7)	0.0544 (8)	−0.0104 (6)	0.0013 (6)	−0.0023 (6)
C16	0.0376 (6)	0.0517 (8)	0.0412 (7)	−0.0162 (6)	0.0049 (5)	−0.0063 (6)
C17	0.0359 (6)	0.0569 (9)	0.0506 (8)	−0.0086 (6)	−0.0060 (6)	0.0013 (7)
C18	0.0387 (6)	0.0420 (7)	0.0496 (8)	−0.0045 (5)	−0.0071 (6)	0.0050 (6)

### Geometric parameters (Å, °)

**Table:**

Cl1—C16	1.7353 (13)	C7—C12	1.3920 (17)
N1—C1	1.3701 (17)	C8—C9	1.3763 (19)
N1—C4	1.3728 (15)	C8—H8	0.9300
N1—C13	1.4216 (15)	C9—C10	1.377 (2)
N2—C5	1.284 (2)	C9—H9	0.9300
N2—C1	1.3556 (16)	C10—C11	1.376 (2)
N3—C6	1.3625 (18)	C10—H10	0.9300
N3—N4	1.3620 (17)	C11—C12	1.3864 (19)
N3—C5	1.372 (2)	C11—H11	0.9300
N4—N5	1.289 (2)	C12—H12	0.9300
N5—N6	1.3550 (19)	C13—C14	1.3846 (19)
N6—C6	1.3252 (18)	C13—C18	1.3818 (18)
C1—C2	1.4018 (16)	C14—C15	1.3832 (19)
C2—C6	1.4153 (17)	C14—H14	0.9300
C2—C3	1.4304 (17)	C15—C16	1.378 (2)
C3—C4	1.3717 (16)	C15—H15	0.9300
C3—C7	1.4696 (16)	C16—C17	1.372 (2)
C4—H4	0.9300	C17—C18	1.3823 (19)
C5—H5	0.9300	C17—H17	0.9300
C7—C8	1.3932 (19)	C18—H18	0.9300
			
C1—N1—C4	107.48 (10)	C9—C8—H8	119.4
C1—N1—C13	128.20 (10)	C7—C8—H8	119.4
C4—N1—C13	123.87 (11)	C8—C9—C10	120.44 (14)
C5—N2—C1	115.46 (12)	C8—C9—H9	119.8
C6—N3—N4	108.86 (13)	C10—C9—H9	119.8
C6—N3—C5	125.13 (12)	C11—C10—C9	119.12 (13)
N4—N3—C5	125.98 (13)	C11—C10—H10	120.4
N5—N4—N3	104.99 (13)	C9—C10—H10	120.4
N4—N5—N6	112.87 (13)	C10—C11—C12	120.94 (14)
C6—N6—N5	105.70 (13)	C10—C11—H11	119.5
N2—C1—N1	123.46 (11)	C12—C11—H11	119.5
N2—C1—C2	128.15 (12)	C11—C12—C7	120.36 (14)
N1—C1—C2	108.37 (11)	C11—C12—H12	119.8
C1—C2—C6	113.98 (11)	C7—C12—H12	119.8
C1—C2—C3	107.62 (10)	C14—C13—C18	120.25 (12)
C6—C2—C3	138.28 (12)	C14—C13—N1	118.84 (11)
C4—C3—C2	105.08 (10)	C18—C13—N1	120.91 (12)
C4—C3—C7	123.84 (11)	C15—C14—C13	119.94 (13)
C2—C3—C7	131.08 (11)	C15—C14—H14	120.0
C3—C4—N1	111.43 (11)	C13—C14—H14	120.0
C3—C4—H4	124.3	C16—C15—C14	119.09 (14)
N1—C4—H4	124.3	C16—C15—H15	120.5
N2—C5—N3	121.32 (13)	C14—C15—H15	120.5
N2—C5—H5	119.3	C17—C16—C15	121.41 (13)
N3—C5—H5	119.3	C17—C16—Cl1	119.38 (12)
N6—C6—N3	107.58 (12)	C15—C16—Cl1	119.21 (12)
N6—C6—C2	136.49 (13)	C16—C17—C18	119.53 (13)
N3—C6—C2	115.89 (12)	C16—C17—H17	120.2
C8—C7—C12	117.85 (12)	C18—C17—H17	120.2
C8—C7—C3	119.35 (11)	C13—C18—C17	119.76 (14)
C12—C7—C3	122.79 (12)	C13—C18—H18	120.1
C9—C8—C7	121.29 (13)	C17—C18—H18	120.1
			
C6—N3—N4—N5	−0.35 (19)	C1—C2—C6—N6	−175.67 (16)
C5—N3—N4—N5	−178.45 (16)	C3—C2—C6—N6	−0.1 (3)
N3—N4—N5—N6	0.2 (2)	C1—C2—C6—N3	1.68 (17)
N4—N5—N6—C6	0.0 (2)	C3—C2—C6—N3	177.24 (14)
C5—N2—C1—N1	−177.25 (13)	C4—C3—C7—C8	−6.91 (19)
C5—N2—C1—C2	1.2 (2)	C2—C3—C7—C8	172.84 (13)
C4—N1—C1—N2	179.69 (12)	C4—C3—C7—C12	171.75 (13)
C13—N1—C1—N2	7.3 (2)	C2—C3—C7—C12	−8.5 (2)
C4—N1—C1—C2	0.98 (14)	C12—C7—C8—C9	0.1 (2)
C13—N1—C1—C2	−171.46 (12)	C3—C7—C8—C9	178.82 (13)
N2—C1—C2—C6	−2.66 (19)	C7—C8—C9—C10	0.1 (2)
N1—C1—C2—C6	175.97 (11)	C8—C9—C10—C11	−0.4 (3)
N2—C1—C2—C3	−179.57 (13)	C9—C10—C11—C12	0.5 (3)
N1—C1—C2—C3	−0.93 (14)	C10—C11—C12—C7	−0.4 (2)
C1—C2—C3—C4	0.51 (14)	C8—C7—C12—C11	0.1 (2)
C6—C2—C3—C4	−175.23 (15)	C3—C7—C12—C11	−178.60 (13)
C1—C2—C3—C7	−179.27 (12)	C1—N1—C13—C14	143.71 (14)
C6—C2—C3—C7	5.0 (3)	C4—N1—C13—C14	−27.59 (19)
C2—C3—C4—N1	0.09 (14)	C1—N1—C13—C18	−37.1 (2)
C7—C3—C4—N1	179.89 (11)	C4—N1—C13—C18	151.55 (14)
C1—N1—C4—C3	−0.67 (15)	C18—C13—C14—C15	−1.2 (2)
C13—N1—C4—C3	172.18 (11)	N1—C13—C14—C15	177.96 (13)
C1—N2—C5—N3	1.2 (2)	C13—C14—C15—C16	1.6 (2)
C6—N3—C5—N2	−2.0 (2)	C14—C15—C16—C17	−0.7 (2)
N4—N3—C5—N2	175.77 (15)	C14—C15—C16—Cl1	179.71 (12)
N5—N6—C6—N3	−0.22 (17)	C15—C16—C17—C18	−0.5 (2)
N5—N6—C6—C2	177.28 (16)	Cl1—C16—C17—C18	179.08 (12)
N4—N3—C6—N6	0.36 (17)	C14—C13—C18—C17	0.0 (2)
C5—N3—C6—N6	178.48 (14)	N1—C13—C18—C17	−179.16 (13)
N4—N3—C6—C2	−177.73 (12)	C16—C17—C18—C13	0.8 (2)
C5—N3—C6—C2	0.4 (2)		

### Hydrogen-bond geometry (Å, °)

**Table:**

*D*—H···*A*	*D*—H	H···*A*	*D*···*A*	*D*—H···*A*
C8—H8···N4^i^	0.93	2.54	3.393 (2)	153
C5—H5···Cl1^ii^	0.93	2.82	3.6045 (15)	143
C12—H12···N6	0.93	2.32	3.191 (2)	155
C18—H18···N2	0.93	2.48	2.979 (2)	114

Symmetry codes: (i) −*x*+1, *y*+1/2, −*z*+3/2; (ii) −*x*+2, *y*−1/2, −*z*+3/2.
